# Atlanta Medical College—Close of the Session

**Published:** 1858-09

**Authors:** 


### EDITORIAL AND MISCELLANEOUS.

### ATLANTA MEDICAL COLLEGE—CLOSE OF THE SESSION.

Simultaneous with this issue of the Journal, will close the fourth Annual Course of Lectures in the Atlanta Medical College.

We can not allow the occasion to pass without saying something in reference to its present position and future prospects.

It is well known to the readers of this Journal, that the Institution has had to encounter from an early period in its history, a most bitter, unscrupulous, and uncompromising opposition from those with whose interest it was supposed to conflict, and then again a more honorable, but perhaps not less decided antagonistic disposition from those who honestly believed that the idea of establishing a Medical School any where out of a large city, was an absurdity, and therefore treated the whole movement with contempt.

There were not, however, wanting a number of prominent and far-seeing gentlemen, members of the Profession and others, who had confidence in the movement, and who bravely "held up the arms" of those who had identified themselves with the enterprise under such discouraging circumstances. The Faculty were composed of a number of gentlemen who had (if no other recommendation) energy and perseverance, and the determination that there should not be a *failure*, if there was *success* in any, and every effort that they could bring to its accomplishment. They determined from its incipiency, that the Institution should be one of high character, and that it should never be justly said that they had lowered or degraded the Profession, but were determined to do all in their power to elevate the standard of requisitions for graduation, and to offer facilities for a high grade of qualification. In accordance with these views, they commenced as pioneers, a system of examinations, or voluntary recitations, which have largely contributed to the unusually practical preparation which has been characteristic of the students of this Institution, this statement being based not simply upon our own observation and experience with those who have graduated here, but upon reliable testimony furnished from other schools to which our students have gone. The Lectures and Course of Instruction altogether in the Institution have been of an eminently practical character, far more so, we have no hesitation in asserting, than in any Medical College of which we have any knowledge, and upon the whole, we think it may be asserted without the fear of successful contradiction, that Students have had better opportunities afforded in the Atlanta Medical College, for preparing to assume the responsible duties of the physician, than is usual—that the Institution has not lowered the requisitions for graduation, and has elevated the standard of qualification—that it has been a decided *success*, not only in the increasing numbers that have annually assembled in her Halls, but *in offering a high order of facilities for obtaining a complete Medical Education.*

In reference to the idea entertained by some, that a successful Medical College could not be established out of a large city, it has been demonstrated, that while in reference to the facilities connected with hospitals, (the advantages of which to the student before graduation, are greatly and designedly overrated,) we cannot compete to the full extent with New York, Philadelphia, or New Orleans, (the latter of which is superior in this respect to any other city in the Union,) there have been found to be some counterbalancing advantages, which lie at the very foundation of the usefulness of the medical man. The medical student has been here recognized as a gentleman, and has been brought into association with the most desirable population, and has occupied such a social position, as to stimulate him to a high regard for public opinion, and to an avoidance of excesses, the indulgence in which too often, during a residence in large cities, lays the foundation for a life of dissipation and degradation—even in this particular, therefore, we do not admit any unfavorable comparison. In the last class we have had students from nearly every Southern State, and among them many gentlemen of education and intelligence, (some of a high order of talent,) a number of whom have attended Medical Institutions located at other points—among them Nashville, Augusta, Philadelphia, New York, New Orleans, Memphis, Richmond, and Charleston, and so far as we are able to learn, the universal testimony is, that the instruction here is more practical, and better adapted to the capacities and *real wants* of the medical student.

This much we say in no boasting spirit, but because we have been constantly forced to defend ourselves from the efforts which have been made from several interested quarters to depreciate the character of the facilities which were furnished here, and because a most unfair and contemptible effort has been made to defame and destroy the Institution, because those who established it, saw fit to select the *Summer,*

(Atlanta being the only city in the South possessing a climate and healthfulness which would adapt it to successful Summer teaching,) as the period for holding its sessions, and for the still more potent reason, that we have the best evidence for believing that the whole attempt to crush the Institution has proceeded from base and sclfish motives, as conclusive evidence of which, is presented the fact, that those who have been most active in the attempt to blast its reputation, and cut the cords of life, were among the first and most earnest to assure us of their friendship, but not profiting by the relation as they had fondly anticipated, have now become the most bitter and uncompromising enemies.

From the same quarter whence the greatest anxiety was manifested during the early period of the existence of the Atlanta Medical College, to establish the most intimate and friendly relations, we recently learn (to use a vulgar term) that the "Triggers are fixed," and the hope of the "*execution*," which, in their imagination, is to take place at the next meeting of the American Medical Association, "is rolled as a sweet morsel under their tongues." We say now, what we should not say, but for conclusive evidence which has been furnished us recently, of a hostile feeling towards us, and a participation in intrigues (which we have long suspected) for our destruction, upon the part of those who have heretofore protested the warmest friendship—until that which was fondly hoped to have been lovingly and successfully nurtured as a "Feeder," has turned out to be a most formidable competitor.

We say now, what we would not have said under other circumstances—if we had been allowed to rest upon our merits, and a fair and honorable course had been pursued by those with whom we are brought into immediate competition—that the Atlanta Medical College is now the *leading* School in the State of Georgia; and in addition to this, has no lower ambition than to attain and adorn an equal position in every particular, with that of any Medical Institution in this broad South. Those who control this College, mean to be, as they have uniformly been, not only fair and just, but liberal and generous, to competitors; but at the same time, it is intended to be distinctly and emphatically understood, that they *demand justice* for themselves, and defy those who, by intrigue, imagine that they have adopted a scheme which will force this Institution to abandon their Summer Sessions, and thus, as they foolishly, but fondly, hope, arrest its tide of prosperity.

It remains but to add, that the Faculty of the Atlanta Medical College desire that their students should attend other Medical Colleges, and that there should be an agreeable and liberal interchange of those who do not attend both courses of lectures in the same Institution; but if circumstances required it, they feel entirely secure of their ability to preserve an independent and honorable position, and will not be deterred by any fear of consequences, from maintaining their rights at all hazards.

The American Medical Association, however, can never, without stultifying itself, and destroying the confidence of the profession—upon which alone it rests for its existence—establish *new requisitions for membership* which have an *ex post facto* operation, and that, too, at the instigation, *and as the result of the base intrigues*, of those who desire to use the influence of this body, for the selfish and dishonorable object of throwing discredit upon competitors who have an equal right to representation with themselves.

In accordance with the high aims and objects which have heretofore prompted those who have controlled the Institution, it has been recently determined to present to those who are seeking a medical education—what has been a *desideratum* in the South. *An Elementary or Preparatory Course*,* distinct and separate from the usual term, embracing a period of four months, commencing with the 1st of November, and ending with the 1st of March, and that, too, without any additional fees to those who become students during the *regular* course, which commences the 1st of May, and ends the 1st of September. In conclusion, we have to say, that the Atlanta Medical College is not only constantly adding to the facilities for medical instruction, but is annually sending out the practical demonstration of her capacity to teach, in those who have been educated in her halls, and is thus laying broad and deep her foundations, and may, therefore, well afford to feel, that if she contended so successfully with her opponents in infancy, and when friends were "few and far between," it is not very likely that the result of efforts now to injure her, will be otherwise than heretofore; and so far from accomplishing the ends designed, it would seem not unreasonable to conclude, will only give a still further impetus to her onward progress.

---

* See Advertisement on back of cover.

